# Written Informed Consent for Computed Tomography of the Abdomen/Pelvis is Associated with Decreased CT Utilization in Low-Risk Emergency Department Patients

**DOI:** 10.5811/westjem.2015.9.27612

**Published:** 2015-11-16

**Authors:** Lisa H. Merck, Laura A. Ward, Kimberly E. Applegate, Esther Choo, Douglas W. Lowery-North, Katherine L. Heilpern

**Affiliations:** * The Warren Alpert Medical School of Brown University, Department of Emergency Medicine and Diagnostic Imaging, Providence, Rhode Island; † Rollins School of Public Health, Department of Biostatistics, Emory University, Atlanta, Georgia; ‡ Emory University School of Medicine, Department of Radiology and Imaging Sciences, Atlanta, Georgia; § The Warren Alpert Medical School of Brown University, Department of Emergency Medicine, Providence, Rhode Island; ¶ Emory University School of Medicine and Grady Memorial Hospital, Department of Emergency Medicine, Atlanta, Georgia

## Abstract

**Introduction:**

The increasing rate of patient exposure to radiation from computerized tomography (CT) raises questions about appropriateness of utilization. There is no current standard to employ informed consent for CT (ICCT). Our study assessed the relationship between informed consent and CT utilization in emergency department (ED) patients.

**Methods:**

An observational multiphase before-after cohort study was completed from 4/2010–5/2011. We assessed CT utilization before and after (Time I/Time II) the implementation of an informed consent protocol. Adult patients were included if they presented with symptoms of abdominal/pelvic pathology or completed ED CT. We excluded patients with pregnancy, trauma, or altered mental status. Data on history, exam, diagnostics, and disposition were collected via standard abstraction tool. We generated a multivariate logistic model via stepwise regression, to assess CT utilization across risk groups. Logistic models, stratified by risk, were generated to include study phase and a propensity score that controlled for potential confounders of CT utilization.

**Results:**

7,684 patients met inclusion criteria. In PHASE 2, there was a 24% (95% CI [10–36%]) reduction in CT utilization in the low-risk patient group (p<0.002). ICCT did not affect CT utilization in the high-risk group (p=0.16). In low-risk patients, the propensity score was significant (p<0.001). There were no adverse events reported during the study period.

**Conclusion:**

The implementation of ICCT was associated with reduced CT utilization in low-risk ED patients. ICCT has the potential to increase informed, shared decision making with patients, as well as to reduce the risks and cost associated with CT.

## INTRODUCTION

Computed tomography (CT) accounts for a significant amount of patient exposure to medical ionizing radiation. Ionizing radiation has been listed by the World Health Organization and the United States Department of Health and Human Services as a carcinogen at low doses.^1^,^2^ Over the last 30 years, the rate of CT use in medical imaging has increased exponentially in the U.S.^3^,^4^ In 1981, approximately 2.3 million CTs were performed in the U.S., and by the year 2006 this number had risen to greater than 60 million.^5^–^7^ CT utilization is estimated to now exceed 80 million per year. Despite the known risk of cancer associated with ionizing radiation, there has been no national standard to educate patients about the risks, benefits, and alternatives to CT imaging, or to obtain patient informed consent for this diagnostic procedure.

In the early 1980s, U.S. citizens were exposed to an estimated 3.6 milliSieverts (mSv) of ionizing radiation per year, 15% of which was attributed to medical sources.^8^,^9^ Less than 25 years later, ionizing radiation exposure has doubled in the U.S., largely attributed to CT and cardiac nuclear medicine imaging. In one large study, Smith-Bindman et al. documented a 7.8% increase in CT utilization annually between 1996 and 2010.^4^ The rapid rise in CT utilization has led to higher patient exposures to radiation, and therefore an increased risk of subsequent cancer development.^10^

A similar acceleration in CT utilization has been documented in U.S. emergency departments (ED), with over a four-fold increase in ED CT utilization documented between 2000–2006.^11^ There is no doubt that CT saves lives and it is often the imaging modality of choice in EDs.^12^,^13^ However, the dramatic increase in CT utilization has raised concerns about the appropriateness of use, cost, and the potential long-term consequences of patient exposure to ionizing radiation.

The practice of informed consent has been uniformly adapted in the U.S. for surgical procedures, lumbar punctures, and even for the administration of intravenous (IV) medications, such as CT contrast.^14^–^18^ However, there is currently no national standard to encourage or require patient informed consent prior to CT imaging.

In a multiphase quality improvement initiative, our research team developed a one-page written informed consent for CT (ICCT) to describe the risks, benefits, and alternatives to CT imaging (Appendix I). The consent protocol was studied at a university hospital over an 18 month period, in an observational multiphase before-after cohort analysis. These data represent the first U.S. study of written informed consent for CT in a large cohort of ED patients.^18^

## METHODS

Investigators developed, piloted, and implemented ICCT during a multiphase before-after cohort study to assess the effect of informed consent on CT utilization. Following the development of the informed consent,^18^ the study was comprised of the following phases:

1. PHASE 1: Characterization of baseline CT utilization in high- and low-risk populations of ED patients presenting for evaluation of abdominal or pelvic pain. Clinical and historical patient risk factors were identified in an a priori fashion; all potential confounders of risk were included in the propensity score model, which was then included in the adjusted, stratified regression model.
2. PHASE 2: Implementation of ICCT, with comparative study of the pre-implementation patient cohort was completed; all study participants were stratified into high and low clinical risk categories. High-risk patients were defined a priori as those demonstrating focal tenderness, rebound tenderness, and/or a rigid abdomen on examination.

The study was completed at a university hospital ED with 37,000 high acuity visits per year. (The hospital serves a quaternary care population consisting of organ transplant, chemotherapy, high-risk neurology and cardiac patients, with a 38% ED hospital admission rate.) The ED serves the local and extended communities, providing emergency care to a culturally and ethnically diverse patient population: in 2013, patient demographics were composed of 50.12 % Black/African American, 48.49% White, and 1.39% other; while 2.38% of patients identified as Hispanic or Latino. ED visits were comprised of a 60:40% female/male patient ratio. The adult hospital provides primary and tertiary care, multiple organ transplant services, and oncological subspecialty care. The study protocol was reviewed and exempted by the university institutional review board, as a quality improvement initiative.

ED patients presenting with abdominal/pelvic pain were selected due to specific characteristics of this patient population: these patients are often alert and able to provide consent; there are accepted alternative imaging modalities to assess abdominal/pelvic pain; and CT leads to ionizing radiation exposure.

### PHASE 1. Demographics, Predictors of CT Utilization, and Predictors of Positive CT Findings

Between April and September 2010, we completed a retrospective chart review to assess baseline utilization of CT and identify criteria associated with significant risk of intra-abdominal or intra-pelvic pathology. Adult patients who presented to the ED with a chief complaint related to abdominal/pelvic pathology and/or who received abdomen/pelvis CT were included. We excluded patients with pregnancy, trauma, or altered mental status.

Research assistants abstracted data from the electronic medical record (EMR) using a standardized tool. Data included demographics; history of immunocompromising illness, cancer, surgery, nausea, vomiting, diarrhea, or vaginal bleeding; physical exam findings of vital signs, abdominal/pelvic tenderness, peritoneal signs, abdominal/pelvic mass, distention; laboratory/imaging diagnostic results; and patient disposition, including observation status, admission, discharge, surgery, and/or intensive care unit admission. We calculated returns to the ED within 30 days, and quantified utilization patterns of ultrasound (US), magnetic resonance imaging (MRI), and inpatient CT. Automated and manually extracted data were matched for accuracy.

### PHASE 2. The Effect of Informed Consent on CT Utilization in High and Low-Risk Patients

In PHASE 2, we conducted an observational cohort study of written informed consent for all eligible ED visits. Adult ED patients with a chief complaint related to abdominal/pelvic pathology or who completed ED CT of abdomen or pelvis were eligible for the study. We excluded patients with pregnancy, trauma, or altered mental status.

An example of the written informed consent is attached in Appendix I. Summary data on patient preferences, the development of the consent, and the quantified educational value of the consent process were published previously. The attached one-page consent form is written at an eighth-grade level and takes approximately one minute to review. Consents are completed with patients by the ordering provider (MD or mid-level provider) and then reviewed by the radiology technician prior to imaging, along with screens for pregnancy, and written consent for IV contrast as appropriate per local standard CT acquisition protocol. The informed consent for CT protocol was active 24 hours a day, seven days a week. Data were abstracted using the methodology outlined in PHASE 1.

### Data Analysis

#### PHASE 1. Demographics, Predictors of CT Utilization, and Predictors of Positive CT Findings

Descriptive statistics were stratified by abdominal/pelvic CT use in ED (Yes/No); univariate analyses (t-test, chi-square) were completed (α=0.05). All distributions of variables were examined and assumptions were met. We constructed a multivariate logistic model to assess CT utilization upon presentation in the ED. Demographics, clinical history, physical exam, and lab results were considered possible predictors. We identified clinically relevant historical, clinical, laboratory, and radiographic variables, a priori (based on past research and clinical acumen) and then applied them to the model. The remainder of the potential predictors were evaluated in the model for statistical significance using stepwise regression and overall model fit using Akaike’s Information Criterion (AIC). All relevant diagnostics were examined and no severe violations were found. This model was validated on random sample patients presenting to the ED at a later time, using both deviance statistics and goodness of fit statistics.

In the cohort of patients who received a CT, additional analyses assessed the predictors of positive CT findings. CT findings were defined as no acute pathological process/negative CT; diverticular disease; appendicitis; obstruction; renal stone; mass; perforation; colitis/inflammation; fluid collection; post-operative changes; biliary tract disease; hernia; other (fibroids, constipation, vascular abnormalities, and/or extra-abdominal findings ie pneumonia). We calculated descriptive statistics and completed univariate analyses (t-test and chi-square). A multivariate logistic model was constructed to define predictors of positive CT (CTP) and negative CT (CTN) using the same methodology described above. This procedure also examined predictors associated with acute versus chronic/negative CT findings. For all analyses, p <0.05 denotes statistical significance. All analyses were completed using SAS software version 9.3.

#### PHASE 2. The Effect of Informed Consent on CT Utilization in High and Low-Risk Patients

We stratified descriptive statistics by CT use in ED. Univariate analyses were completed. Using the multivariate logistic regression model generated from the baseline data, we assessed patterns of ED CT utilization for one year (April 2010 – May 2011).

In the analysis, study participants were stratified into high and low clinical risk categories. High-risk patients were defined as those demonstrating focal tenderness, rebound tenderness, and/or a rigid abdomen on examination. We created logistic regression models examining the relationship of ED CT use and the use of informed consent, stratifying by high- or low-risk status. The following independent potential confounders were chosen for the models based on statistical significance (p-value <0.05) and/or clinical relevance: age, sex, initial pain score, immunocompromised state; history of nausea, vomiting, and/or cancer; presence of mass, distention, bowel sounds, and/or vaginal bleeding; laboratory data including white blood cell count and urine nitrites, as well as temperature and systolic blood pressure. We used these variables to create a propensity score for inclusion in the final model. When included in the regression model, the propensity score is a measure of the relationship between receiving an ED CT, clinical and radiographic findings, and the intervention of informed consent. The propensity score controls for potential confounders, without the inflated standard errors that often arise from controlling for a large number of individual confounders and helps to balance the differences that may exist between groups in an observational study, providing estimates closer to the true treatment effect. The logistic regression represents overall CT utilization after adjustment for all clinically relevant confounding variables via the propensity score. This is distinct from the unadjusted values. We examined all relevant diagnostics and found no severe violations.^19^,^20^

A sensitivity analysis of the missing data was completed for this model, comparing the complete case analysis to an analysis using multiple imputation to control for missing data. No significant differences were found.^19^,^20^ We completed all analyses using SAS software version 9.3.

## RESULTS

### PHASE 1. Demographics, Predictors of CT Utilization, and Predictors of Positive CT Findings

#### CT Utilization

We identified 4,702 patients as presenting to the ED with a chief complaint of abdominal or pelvic pathology; 4,108 met eligibility criteria. Thirty-two percent of these patients (n=1,333) received CT (CTED). The CTED group demonstrated several significant differences from patients who did not receive CT (nCTED). CTED patients had higher initial pain scores (6.9 vs 5.5, p<0.001) and were older (50.4 years vs 48.5 years, p=0.003) than nCTED patients. CTED patients were more likely to endorse history of nausea (p<0.001) or vomiting (p=0.006). Patients with history of cancer, compromised immune systems, or vaginal bleeding were less likely to receive CTED (p<0.001). While patients with history of cancer or immunocompromise received fewer CT studies, this subset demonstrated more frequent total positive CT findings (p<0.001). Patients who received ED US or MRI were less likely to receive ED CT (p<0.001). Significant physical exam and laboratory findings are further reported in Table 1.

In summary, factors associated with CT utilization in PHASE I included age and immune competence; symptoms of nausea, vomiting, and pain; elevation in serum alanine aminotransferase (ALT), hematocrit, and white blood cells (WBC); and physical exam findings of focal or rebound tenderness (Table 2). Patients undergoing active chemotherapy therapy were noted to more frequently receive medical management in the ED independent of CT imaging. The use of MRI and US were inversely correlated with ED CT utilization.

#### CT Findings

Thirty-two percent of participants (1,333/4,108) received CT, and 74% (n=985) were noted to have positive CT imaging (CTP). All CT-specific chart data were complete. CTP patients were more likely than CT negative (CTN) patients to be older or to report nausea, history of immunocompromise, cancer, palpable mass, distention, elevated serum lipase, bilirubin, WBC, and/or prior surgery (p<0.025). In the multivariate model, CTP patients more frequently demonstrated history of cancer, immunocompromised state, and/or prior surgery (p<0.025).

CT results were stratified into two groups, those with acute pathology (CTPA, 45% n=594) and those with chronic pathology and/or negative findings (CTPC, 55% n=739). CTPA patients were more likely to have a history of nausea or recent surgery, and to demonstrate abnormal lab values for lipase, bilirubin, and WBC (p<0.05). Statistically significant physical exam predictors for CTPA in an adjusted model included sex and the laboratory finding of elevated white blood count.

### PHASE 2. The Effect of Informed Consent on CT Utilization in High and Low-Risk Patients

There were 7,684 patients who met inclusion criteria for analyses in the study of CT utilization before and after implementation of ICCT. Of these, 4,108 were included before implementing the informed consent (PHASE 1) and 3,576 patients were included after implementing informed consent (PHASE 2). There were no significant differences in patient demographics for sex or ethnicity between the two study phases. All patients from PHASE 1 and PHASE 2 were treated as independent. Unadjusted data illustrate that 32% of patients in PHASE 2 received CTED, 21% of the low-risk group received CTED and 47% of the high-risk group. This compares to 32% of patients in PHASE 1 receiving CTED, 22% of the low-risk group and 48% of the high-risk group.

Of the 3,576 patients studied after implementing informed consent, those who received CT in the ED were noted to be older (<0.001) and reported higher pain scores (<0.001) than nCTED patients. CTED patients were more likely to present with focal tenderness, rebound tenderness, mass, distention, decreased bowel sounds and nausea (p<0.05). History of immunocompromise and/or presentation with fever, diarrhea, or vaginal bleeding were negatively correlated with CTED. MRI and US use were negatively correlated with CTED (Table 3).

Patients seen during PHASE 1 were older than patients seen during PHASE 2 (49.1 v. 47.3, p-value<0.001). Race and sex were similar between PHASE 1 and PHASE 2 (Table 4). Participants who received a CT after ICCT, were more likely to present with peritoneal signs, focal tenderness, history of vomiting, decreased bowel sounds, mass, and/or history of cancer than those who received a CT before ICCT (p<0.05).

There were 3,130 patients in the high-risk group and 4,554 patients in the low-risk group. Of those in the high-risk group, 1,497 (47.8%) had a CT performed and 1,113 (74.3%) of these patients had a positive CT result. CTs completed in these high-risk patients made up 35.6% of the total positive CTs performed. Of those in the low-risk group, 1,004 (22.0%) had a CT performed and 740 (73.7%) had a positive result; positive CT scans in the low-risk group represent a value of 16.2% of the total CTs performed.

After implementation of the ICCT protocol there was a 24% (95% CI [10–36%]) reduction in CT utilization in the low-risk patient population (p=0.002) after controlling for clinical confounders via the propensity score. The ICCT protocol did not affect utilization in the high-risk population (p=0.16) after controlling for the propensity score. The propensity score was statistically significant for the low-risk group (p=0.002), indicating the set of variables included in the propensity score were statistically related to the performance of ED CT.

There were no adverse events reported or identified during the study period. Returns to the ED within 30 days of the initial visit were not different between risk groups, before or after implementation of ICCT (p=0.87). CT utilization in the clinically high-risk population did not change during the study period. Multiple imputation analyses were performed and did not differ from the complete data analysis; no bias was identified. Patients who received CT in the ED were more likely to receive CT during their inpatient stay in the hospital, when compared to patients who did not receive ED CT. Results of the stratified regression models are available in Table 4.

## DISCUSSION

In this novel, multiphase study, investigators developed the ICCT tool and demonstrated its feasibility, acceptability, and ability to improve patient knowledge about the risks of ionizing radiation from CT.^18^ Researchers identified factors that placed patients at high and low risk for clinically important findings on CT. ICCT implementation was associated with reduced CT use in low-risk patients, and did not affect CT utilization in the high-risk patient population.

In this cohort study, the derived propensity score adjusts for clinically relevant covariates, such as laboratory, historical, and physical exam findings related to patient course and evaluation. Such variables are present in the cohorts, but are not presumed to be balanced between groups. The unadjusted data do not account for such covariates and do not illustrate statistically significant differences between groups. However, the propensity model controls for the clinically relevant confounders, and illustrates a significant difference between PHASE 1 and PHASE 2 in CT utilization, within the low-risk patient population.

The ICCT protocol is intended to engage patients in shared medical decision-making. This approach has the potential to help physicians achieve national goals to reduce unnecessary medical imaging and resource use, as supported by the 24% (95% CI [10–36%]) reduction we found in CT utilization among low-risk patients after implementation of the protocol.^21^

Although our study is limited by secular trends in CT utilization and physician education via the informed consent tool; it is essential to note that reduction in CT utilization was only noted in the low-risk patient population of our cohort; no significant change in utilization occurred in high-risk patients. This relationship supports future application of informed consent as an educational and shared decision-making tool.

Much of the data on carcinogenesis from ionizing radiation have been derived from animal models or retrospective studies of nuclear workers/atomic bomb survivors. In 2012, Pearce et al. published the first longitudinal cohort study to identify a linear dose response curve between CT exposure and cancer development.^10^ Pearce et al. call for action to decrease radiation exposure related to CT to the lowest possible dose (ALARA, “as low as reasonably achievable”), and to perform scans only when clearly justified. Using the linear no threshold model, routine abdominal-pelvic CT is conservatively estimated to induce fatal cancer in 1 per 5,000–10,000 patients exposed.^22^,^34^–^37^

Despite the known risks of ionizing radiation exposure, it is neither standard care nor routine practice to obtain informed consent for CT.^17^,^18^ This may be due to the fact that ionizing radiation from CT causes no immediately tangible effect or visible scar and the resulting development of cancer may not occur for decades. However, like surgery, ionizing radiation leaves a biological mark on the patient. Furthermore, the calculated risk of cancer induction from CT is surprisingly greater than the risk associated with other medical interventions (e.g. blood transfusion) that routinely require hospital regulated informed consent.^18^ As blood transfusion carries an estimated 1 in 250,000 risk of infection with hepatitis C virus, and a 1 in 1.3 million risk of acquiring HIV,^38^ it appears we have failed to proportionally perform due diligence with regard to informed consent for CT.

Whether communication with the patient about the benefits and risks of CT should be through informed consent or via a shared decision-making process has been an ongoing debate.^18^,^39^,^40^ Yet, without informed consent, there is no evidence that information is being shared with patients with any frequency or in a standardized fashion.

The study’s ICCT process is a rapid, practical, and effective method to educate patients about the risks, benefits, and alternatives to CT imaging. Over the extended cohort, ICCT was associated with a reduction of CT utilization in low-risk ED patients who presented with abdominal/pelvic pain. Future analyses will assess the effect of a video educational module/written informed consent on the utilization patterns of CT, MRI, and US in the ED population.

## LIMITATIONS

Investigators were not able to control for institutional and secular trends within the longitudinal cohort that may have affected CT utilization. However, the effect of informed consent was observed to be limited to the low-risk patients within the cohort, and not observed in all ED patients.

Our study cannot discriminate between the effect of provider versus patient education on utilization, as these occurred simultaneously. The overall act of instituting ICCT was significantly associated with reduced utilization, increased patient understanding, and positive patient preferences.

Additional limitations include the fact that chart data were collected via retrospective review of the EMR within a single center. It is unknown whether or not a patient visited the ED multiple times within or between the phases, all patient visits were treated as independent interactions for statistical purposes. However, several features strengthen the likelihood that our findings are relevant to a range of ED settings. As a collaborative, multidisciplinary effort, the protocol was validated by a variety of stakeholders, including physician/nursing colleagues, radiation physicists, technicians, medical ethicists, and patient consultants. Furthermore, the study’s ED population was racially, ethnically, and socioeconomically diverse, making the study likely to be relevant to many different practice settings. Finally, with a brief, <1-minute script, the protocol was designed to be minimally disruptive to the practice setting.

While the use of the propensity score adjusted for potential unbalanced characteristics between groups and controlled inflated standard errors, propensity scores, by their nature, are unable to control for unmeasured potential confounders.

## CONCLUSION

The implementation of informed consent for CT was associated with reduced CT utilization, after controlling for clinical confounders, in this large prospective cohort study of ED patients. No significant adverse events or complications were reported throughout the study. ICCT has the potential to increase informed, shared decision-making for patients, as well as to reduce the risks and costs associated with the CT procedure.

## Supplementary Information



## Figures and Tables

**Table 1:** Demographics and clinical characteristics of patients prior to implementation of informed consent protocol, stratified by ED prescription of CT imaging (n=4,108).

	Overall^a^(n=4,108)	ED CT(n=1,333)	No ED CT(n=2,775)	p-value^b^
Demographics				
Average age†	49.14 (19.00)	50.42 (18.70)	48.53 (19.11)	0.003
Sex†‡				
Male	1,610 (39.19%)	506 (37.96%)	1,104 (39.78%)	0.26
Female	2,498 (60.81%)	827 (62.04%)	1,671 (60.22%)	
Ethnicity				0.005
Black	1,640 (40.22%)	488 (36.86%)	1,152 (41.83%)	
White	2,135 (52.35%)	741 (55.97%)	1,394 (50.62%)	
Other	303 (7.43%)	95 (7.18%)	208 (7.56%)	
Labs				
ALT (n=3,538)	33.00 (71.02)	35.42 (96.18)	31.64 (51.74)	0.19
AST (n=3,546)	40.38 (80.22)	43.48 (120.00)	38.66 (44.14)	0.17
Creatinine (n=3,730)	1.26 (1.57)	1.25 (1.56)	1.26 (1.57)	0.83
Hematocrit (n=3,735)†	36.85 (5.98)	37.71 (5.74)	36.38 (6.06)	<0.001
Lactic acid (n=308)	2.07 (1.73)	2.07 (1.70)	2.08 (1.77)	0.99
Lipase (n=1,752)†‡	42.48 (148.59)	37.59 (90.41)	46.20 (180.60)	0.19
Total bilirubin (n=3,539)†‡	1.03 (2.23)	1.03 (2.24)	1.03 (2.23)	0.93
WBC (n=3,731)†‡				
>11.1	904 (22.0%)	379 (28.45)	525 (18.9%)	<0.001
<3.6	199 (4.8%)	44 (3.3%)	155 (5.6%)	
Vital signs				
Systolic blood pressure (n=4,104)†				
<100 or >160mmHg	675 (16.5%)	265 (19.9%)	410 (14.8%)	<0.001
Diastolic blood pressure (n=4,108)	76.96 (13.79)	78.05 (14.71)	76.44 (13.29)	<0.001
Heart rate (n=4,100)	88.03 (18.56)	86.83 (18.37)	88.61 (18.62)	0.004
Body temperature (n=4,106)	36.84 (0.70)	36.78 (0.61)	36.87 (0.74)	<0.001
Signs and symptoms				
Initial pain score (n=4,107)	5.93 (3.60)	6.86 (3.19)	5.48 (3.70)	<0.001
Focal tenderness‡	1,570 (38.22%)	762 (57.16%)	808 (29.12%)	<0.001
Abdomen soft	4,094 (99.66%)	1,323 (99.25%)	2,771 (99.86%)	0.003^c^
Rebound tenderness‡	71 (1.73%)	48 (3.60%)	23 (0.83%)	<0.001
Bowel sounds	4012 (97.66%)	1,276 (95.72%)	2,736 (98.59%)	<0.001
Mass (n=3,988)†	59 (1.48%)	31 (2.38%)	28 (1.04%)	0.001
Distention (n=4,004)†	219 (5.47%)	99 (7.59%)	120 (4.44%)	<0.001

	Overall^a^(n=4,108)	ED CT(n=1,333)	No ED CT(n=2,775)	p-value^b^
Medical history				
Immunocompromise^d^†	614 (14.95%)	142 (10.65%)	472 (17.01%)	<0.001
Cancer†	670 (16.31%)	179 (13.43%)	491 (17.69%)	<0.001
Blood in urine (n=3,303)†	1,233 (37.33%)	435 (36.52%)	798 (37.78%)	0.47
Leukocytes in urine (n=4,102)	1,364 (41.30%)	470 (39.46%)	894 (42.33%)	0.11
Nitrites in urine (n=4,101)	206 (6.24%)	71 (5.96%)	135 (6.39%)	0.62
Fever (n=4,102)^e^	730 (17.80%)	192 (14.44%)	538 (19.41%)	<0.001
Nausea (n=4,101)†‡	2,149 (52.40%)	814 (61.20%)	1,335 (48.18%)	<0.001
Vomiting (n=4,102)	1,443 (35.18%)	507 (38.12%)	936 (33.77%)	0.006
Diarrhea (n=4,102)	628 (15.31%)	191 (14.360%)	437 (15.76%)	0.24
Vaginal bleeding^a^ (n=4,102)	96 (2.34%)	9 (0.68%)	87 (3.14%)	<0.001
Surgery 1–30 days ago†‡	266 (6.48%)	105 (7.88%)	161 (5.80%)	0.011
Surgery 31–60 days ago	91 (2.22%)	36 (2.70%)	55 (1.98%)	0.14
Surgery 61–90 days ago	77 (1.87%)	20 (1.50%)	57 (2.05%)	0.22
Surgery 91–365 days ago†	343 (8.35%)	122 (9.15%)	221 (7.96%)	0.20

*ED,* emergency department; *CT*, computerized tomography; *ALT*, alanine aminotransferase; *AST*, aspartate transaminase; *WBC*, white blood cell

† These variables were also significantly related to positive CT findings (CTP).

‡ These variables were also significantly related to acute CT findings (CTPA).

a Statistics provided are mean (std dev) for continuous and discrete variables and n (%) for categorical variables.

b p-values are results of t-tests or chi-square tests.

c Used Fisher’s exact test due to small cell counts.

*ED,* emergency department; *CT*, computerized tomography

† These variables were also significantly related to positive CT findings (CTP).

‡ These variables were also significantly related to acute CT findings (CTPA).

a Categorized as yes, no, n/a.

d Patients with active chemotherapy or immunomodulatory therapy were included in this cohort.

e Patients with symptoms of gastroenteritis (fever + symptoms of vomiting/diarrhea) were included in this cohort.

**Table 2:** Predictors of ED prescription of CT imaging prior to implementation of informed consent in multivariable logistic model.

	Beta	Std error	p-value	Adjusted odds ratio	Adjusted OR 95% CI
Intercept	2.44	2.43	n/a	n/a	n/a
Presence of bowel sounds	−0.44	0.14	0.0012	0.41	(0.24, 0.70)
Focal tenderness	0.492	0.04	<0.0001	2.67	(2.25, 3.18)
Immune compromised	−0.274	0.06	<0.0001	0.58	(0.45, 0.74)
Nausea	0.148	0.06	0.0085	1.34	(1.08, 1.67)
Rebound tenderness	0.436	0.16	0.0076	2.39	(1.26, 4.55)
Soft abdomen	−1.07	0.48	0.0255	0.12	(0.02, 0.77)
History of vaginal bleeding	−0.61	0.23	0.0072	0.30	(0.12, 0.71)
History of vomiting	−0.20	0.06	0.0004	0.68	(0.54, 0.83)
ED MRI	−0.95	0.41	<0.0001	0.39	(0.17, 0.87)
ED ultrasound	−0.57	0.14	<0.0001	0.55	(0.43, 0.74)
ALT	0.01	0.001	0.0229	1.001	(1.000, 1.003)
Hematocrit	0.03	0.01	<0.001	1.02	(1.01, 1.05)
White blood cell count	0.05	0.01	<0.0001	1.05	(1.03, 1.07)
Temperature	−0.13	0.06	0.0437	0.88	(0.78, 0.99)
Age on arrival	0.02	0.002	<0.0001	1.02	(1.01, 1.02)
Initial pain score	0.09	0.01	<0.0001	1.10	(1.07, 1.12)

*ED*, emergency department; *CT*, computerized tomography; *OR*, odds ratio; *CI*, confidence interval; *MRI*, magnetic resonance imaging; *ALT*, alanine aminotransferase

**Table 3:** Demographics and clinical characteristics of patients after implementation of informed consent protocol, stratified by ED prescription of CT imaging (n=3,576).

	Overall^a^ (n=3,576)	ED CT (n=1,168)	No ED CT (n=2,404)	p-value^b^
Demographics				
Age	47.28 (18.68)	49.81 (18.02)	46.06 (18.87)	<0.001
Sex				
Male	1,442 (40.32%)	482 (41.27%)	960 (39.87%)	0.42
Female	2,134 (59.68%)	686 (58.73%)	1,448 (60.13%)	
Ethnicity				0.06
Black	1,471 (41.53%)	451 (39.12%)	1,020 (42.70%)	
White	1,803 (50.90%)	620 (53.77%)	1,183 (49.52%)	
Other	268 (7.49%)	82 (7.02%)	186 (7.73%)	
Labs				
ALT (n=3,211)	31.42 (53.84)	30.85 (46.93)	31.73 (57.23)	0.64
AST (n=3,212)	39.67 (67.74)	41.21 (84.63)	38.83 (60.21)	0.40
Creatinine (n=3,333)	1.25 (1.60)	1.18 (1.24)	1.28 (1.76)	0.06
Hematocrit (n=3,325)	37.48 (6.20)	38.31 (5.61)	37.04 (6.45)	<0.001
Lactic acid (n=315)	1.91 (1.67)	2.02 (1.96)	1.79 (1.30)	0.22
Lipase (n=1,933)	42.11 (119.9)	44.12 (118.2)	40.77 (120.9)	0.55
Total bilirubin (n=3,212)	0.99 (1.63)	0.99 (1.41)	0.99 (1.74)	0.97
WBC (n=3,324)				
High	849 (25.5%)	372 (32.2%)	477 (22.0%)	<0.001
Low	130 (3.9%)	27 (2.3%)	103 (4.8%)	
Vital signs				
Systolic blood pressure (n=3,574)				
Abnormal	144 (4.0%)	44 (3.8%)	100 (4.2%)	0.5798
Diastolic blood pressure (n=3,573)	69.60 (17.78)	69.04 (18.39)	69.88 (17.47)	0.20
Heart rate (n=3,574)	83.33 (19.57)	81.84 (20.29)	84.05 (19.17)	0.002
Body temperature (n=3,572)	36.34 (0.75)	36.17 (0.77)	36.42 (0.73)	<0.001
Signs and symptoms				
Initial pain score (n=3,575)	6.11 (3.53)	6.99 (3.05)	5.67 (3.66)	<0.001
Focal tenderness	1,526 (42.67%)	720 (61.64%)	806 (33.47%)	<0.001
Soft abdomen	3,559 (99.52%)	1,160 (99.32%)	2,399 (99.63%)	0.205
Rebound tenderness	81 (2.27%)	58 (4.97%)	23 (0.96%)	<0.001
Bowel sounds	3,408 (95.30%)	1059 (90.67%)	2349 (97.55%)	<0.001
Mass (n=3,988)	91 (2.54%)	61 (5.22%)	30 (1.25%)	<0.001
Distention (n=4,004)	188 (5.26%)	89 (7.62%)	99 (4.11%)	<0.001

	Overall^a^ (n=3,576)	ED CT (n=1,168)	No ED CT (n=2,404)	p-value^b^
Medical history				
Immunocompromised	551 (15.41%)	150 (12.84%)	401 (16.66%)	0.003
Cancer	632 (17.67%)	208 (17.81%)	424 (17.61%)	0.88
Blood in urine (n=2,981)^c^	1,075 (36.06%)	361 (33.64%)	714 (37.42%)	0.04
Leukocytes in urine (n=2,981)	1,229 (41.235)	386 (35.97%)	843 (44.18%)	<0.001
Nitrites in urine (n=2,981)	157 (5.27%)	33 (3.08%)	124 (6.50%)	<0.001
Fever	513 (14.35%)	148 (12.67%)	365 (15.16%)	0.05
Nausea	2,059 (57.58%)	734 (62.84%)	1,325 (55.02%)	<0.001
Vomiting	1,452 (40.60%)	491 (42.04%)	961 (39.91%)	0.22
Diarrhea	595 (16.64%)	153 (13.10%)	442 (18.36%)	<0.001
Vaginal bleeding^d^	94 (2.63%)	7 (0.60%)	87 (3.61%)	<0.001

*ED*, emergency department; *CT*, computerized tomography; *ALT*, alanine aminotransferase; *AST*, aspartate transaminase; *WBC*, white blood cell

a Statistics provided are mean (std dev) for continuous and discrete variables and n (%) for categorical variables.

b p-values are results of t-tests or chi-square tests.

*ED*, emergency department; *CT*, computerized tomography

a Statistics provided are mean (std dev) for continuous and discrete variables and n (%) for categorical variables.

b p-values are results of t-tests or chi-square tests.

c Patients with nephrolithiasis and/or UTI are included in this cohort.

d Categorized as yes, no, n/a.

**Table 4:** Model results of effect of informed consent on ED prescription of CT imaging stratified by risk status.

	Low risk (n=3783)			High risk (n=2877)		
	
	Adjusted OR	95% CI	P-value	Adjusted OR	95% CI	P-value
Intercept	--	--	--	--	--	--
Time 2	0.76	(0.64, 0.90)	0.002	0.89	(0.76, 1.05)	0.17
Propensity score	1.74	(1.23, 2.45)	0.002	0.89	(0.62, 1.29)	0.54

*ED*, emergency department; *CT*, computerized tomography; *OR*, odds ratio; *CI*, confidence interval
