# Supplementary material for: Written Informed Consent for Computed Tomography of the Abdomen/Pelvis is Associated with Decreased CT Utilization in Low-Risk Emergency Department Patients
Source: West J Emerg Med. 2015 Nov 16;16(7):1014–24. doi: 10.5811/westjem.2015.9.27612 (PMC4703183; doi:10.5811/westjem.2015.9.27612)
Supplement: Supplementary file 1 [file wjem-16-1014-s001.pdf]

Patient Consent for Computerized Tomography (CT) Scan
At Emory University Hospital

Date: ____/_____/________ Time: __________ Room Number: ________

Diagnosis: ____

CT scan (circle): **Abdomen** **Pelvis** **Abdomen + Pelvis** **With or Without IV Contrast**

What are the benefits of having a Computerized Tomography [CT] scan?
Your doctor has recommended that you have a CT (or "CAT") scan to evaluate __________________________. CT scans give doctors detailed pictures of the body that are used to diagnose illness and plan treatment. In many cases, CT scans detect illness that is very hard to diagnose with other tests. If your doctor knows you have an illness, he or she may use a CT scan to decide on the best therapy for your illness. Your doctor believes that the benefits of the CT scan outweigh the risks. Your doctor will talk about the benefits of the CT scan with you. You may also ask your doctor about options other than CT scanning (these options are discussed further on page 2 in the section labeled "Alternatives").

What are x-rays and CT scans?
An x-ray uses invisible beams of energy (called *radiation*) to make pictures of organs in your body. This energy is absorbed by tissue in the body such as in the bones, lungs, thyroid, and heart. The energy is absorbed by different parts of the body to create an image. A CT scan also uses *radiation.* CT scanners take many thin x-rays to create even more detailed images. The x-rays come from a camera that is rotated in a circle around the body. The x-rays are used to make pictures of the inside of the body.

CT scans give your doctor much more information than a single x-ray. However, CT uses more *radiation,* because many x-rays are used to create the images.

What are the risks of having x-rays and CT scans?
CT scans, like x-ray, use radiation energy. This type of radiation occurs naturally. Every day, we are exposed to small amounts of radiation in our environment. This type of radiation is called *background radiation*. Background radiation is measured in *millisieverts* (mSv). To understand the amount of radiation exposure in a CT, we compare the radiation dose in a CT scan to the background radiation dose. The table below compares the radiation in a CT scan to the amount of time it would take to receive that same amount of radiation in the environment.

Radiation Type	Effective Radiation Dose	Amount of Background Radiation	Cancer Risk
Background	3 mSv per year		
1 view chest x-ray	0.1 mSv	1 day	
Abdominal CT	7-20 mSv[3, 4]	28-80 months	1 per 5,000-10,000 patients[3, 4]

Approximate ranges in the table represent differences due to CT machine, patient age, and patient body weight

On average, a person in the United States is exposed to 3 mSv of radiation every year. Any kind of radiation can damage the cells in your body. Small exposures do not cause any painful or visible damage when they

occur. Large or repeated exposure to radiation may harm some of the cells in your body. In time, these damaged cells may change and become cancer cells.

In our society, forty percent of all people develop cancer in their lifetime. CT scans are linked with a very small increase in this baseline risk of developing cancer. We can also compare risk of CT to other risks people take every day. The risk of getting cancer from an abdominal CT scan is about the same as the risk of a getting in a car accident from driving 10,500-30,000 miles. The increased cancer risk from CT scan exposure is small, but it exists, so you need to be aware of this risk. The more radiation a person is exposed to, the higher their risk of cancer. The amount of radiation used for CT scans is controlled carefully. The smallest possible dose is given to each patient in order to produce a high quality CT scan. However, there is a small amount of radiation exposure every time you get a CT scan.

Alternatives: Sometimes, alternative tests other than CT scanning may be used to evaluate your illness. For example, you may be observed over time in the hospital. Other tests may help to diagnose your illness, such as ultrasound (which uses sound waves) or MRI (which uses magnetic resonance imaging). Sometimes, these studies may help your doctor. However, in many cases CT creates the best images to diagnose an illness.

Summary:

Your doctor has recommended a CT scan to evaluate your illness. If you choose not to have the CT scan, there is a risk of missing a serious or even life threatening illness. There is a risk of death or disability if an underlying illness is not found. CT is a procedure, and like surgery, it has both benefit and risk. The benefit of the procedure is that it may help to diagnose an underlying condition that otherwise may not be identified. The risk of a CT scan is exposure to radiation. This radiation exposure may have a small increased risk of developing cancer in the future. It is important to discuss any concerns that you may have about these risks with your physician.

It is important for you to understand the risk of CT scans and to consent if you wish to have a CT scan. Please ask your doctor if you have any other questions or concerns about your CT scan.

I understand the risks and benefits of this procedure. My healthcare provider has answered my questions. I would like to (check one):

Give consent for CT at this time___________ **Decline CT at this time ______________________**

Printed Name of Patient_____________________ **Signature of Patient ___________________________**

Date: ___/___/_______

Printed Name of Healthcare Provider Obtaining Consent: ____

Signature of Healthcare Provider Obtaining Consent: ____

Date: ___/___/_______, Time:_______________

Printed Name of Technologist Reviewing Consent Form: ____

References:

1. http://www.imagingpathways.health.wa.gov.au/includes/index.html
2. The Image Gently Campaign: www.imagegently.org and http://www.pedrad.org/associations/5364/files/ImageGently 8.5x11 Brochure.pdf
3. BEIR VII Public Summary from the National Academy of Sciences. 2005. http://books.nap.edu/openbook.php?record_id=11340&page=1
4. Smith-Bindman R et al. "Radiation Dose Associated with Common Computed Tomography Examinations and the Associated Lifetime Attributable Risk of Cancer." Arch Intern Med. Vol. 169 (No. 22); page 2078-2086. 14/28 Dec 2009.
